# APAtizer: a tool for alternative polyadenylation analysis of RNA-Seq data

**DOI:** 10.1093/bioinformatics/btae689

**Published:** 2024-11-18

**Authors:** Bruno Sousa, Maria Bessa, Filipa L de Mendonça, Pedro G Ferreira, Alexandra Moreira, Isabel Pereira-Castro

**Affiliations:** Gene Regulation Group, i3S—Instituto de Investigação e Inovação em Saúde, Universidade do Porto, Porto, 4200-135, Portugal; IBMC—Instituto de Biologia Molecular e Celular, Universidade do Porto, Porto, 4200-135, Portugal; FCUP—Faculdade de Ciências, Universidade do Porto, Porto, 4169-007, Portugal; Gene Regulation Group, i3S—Instituto de Investigação e Inovação em Saúde, Universidade do Porto, Porto, 4200-135, Portugal; IBMC—Instituto de Biologia Molecular e Celular, Universidade do Porto, Porto, 4200-135, Portugal; Gene Regulation Group, i3S—Instituto de Investigação e Inovação em Saúde, Universidade do Porto, Porto, 4200-135, Portugal; IBMC—Instituto de Biologia Molecular e Celular, Universidade do Porto, Porto, 4200-135, Portugal; FCUP—Faculdade de Ciências, Universidade do Porto, Porto, 4169-007, Portugal; Laboratory of Artificial Intelligence and Decision Support, Institute for Systems and Computer Engineering Technology and Science, Porto, 4200-465, Portugal; Gene Regulation Group, i3S—Instituto de Investigação e Inovação em Saúde, Universidade do Porto, Porto, 4200-135, Portugal; IBMC—Instituto de Biologia Molecular e Celular, Universidade do Porto, Porto, 4200-135, Portugal; ICBAS—Instituto de Ciências Biomédicas Abel Salazar, Universidade do Porto, Porto, 4050-313, Portugal; Gene Regulation Group, i3S—Instituto de Investigação e Inovação em Saúde, Universidade do Porto, Porto, 4200-135, Portugal; IBMC—Instituto de Biologia Molecular e Celular, Universidade do Porto, Porto, 4200-135, Portugal

## Abstract

**Summary:**

APAtizer is a tool designed to analyze alternative polyadenylation events on RNA-sequencing data. The tool handles different file formats, including BAM, htseq, and DaPars bedGraph files. It provides a user-friendly interface that allows users to generate informative visualizations, including Volcano plots, heatmaps, and gene lists. These outputs allow the user to retrieve useful biological insights such as the occurrence of polyadenylation events when comparing two biological conditions. In addition, it can perform differential gene expression, gene ontology analysis, visualization of Venn diagram intersections, and correlation analysis.

**Availability and implementation:**

Source code and example case studies for APAtizer are available at https://github.com/GeneRegulationi3S/APAtizer/.

## 1 Introduction

Alternative polyadenylation (APA) is a co-transcriptional mechanism that occurs in approximately 70% of human genes, producing mRNA isoforms by the recognition of alternative polyadenylation signals (PAS) (Hoque *et al.* 2013). Alternative polyadenylation that occurs in 3ʹ untranslated regions (3ʹUTR-APA) has a major role in gene expression regulation due to the presence of *cis*-regulatory elements in the 3ʹUTR that influence the mRNA stability, mRNA localization, and production of the resulting protein (Mayr 2017). 3ʹUTRs often serve as a binding platform for microRNAs and RNA-binding proteins, which affect the fate of the mRNA transcript (Pereira-Castro and Moreira 2021). Therefore, long 3ʹUTRs contain more *cis*-regulatory elements than short 3ʹUTRs (Burri and Zavolan 2021). Genome-wide studies have shown that certain 3ʹUTR profiles are characteristic of specific tissues and diseases, further proving the role of APA in gene expression regulation (Pereira-Castro and Moreira 2021). It is recognized that cancer cells tend to produce mRNAs with shorter 3ʹ untranslated regions (3ʹUTRs) than normal cells, as a result of 3ʹUTR-APA (Mayr and Bartel 2009). In addition, some tumor suppressor genes produce truncated mRNAs by intronic polyadenylation (IPA) with the potential of hampering their functions (Lee *et al.* 2018). These results indicate that there is a correlation between APA and cancer.

To facilitate the analysis of APA events in RNA-seq data we have developed APAtizer. This tool is able to perform 3ʹUTR-APA and/or IPA analysis based on two algorithms widely used for APA analysis: APAlyzer (Wang and Tian 2020) and DaPars (Feng *et al.* 2018, Li *et al.* 2021). APAtizer thus represents an innovative user-friendly and freely available resource poised to facilitate the analysis of extensive RNA-Seq datasets from multiple sources.

## 2 Description

**Figure 1.**
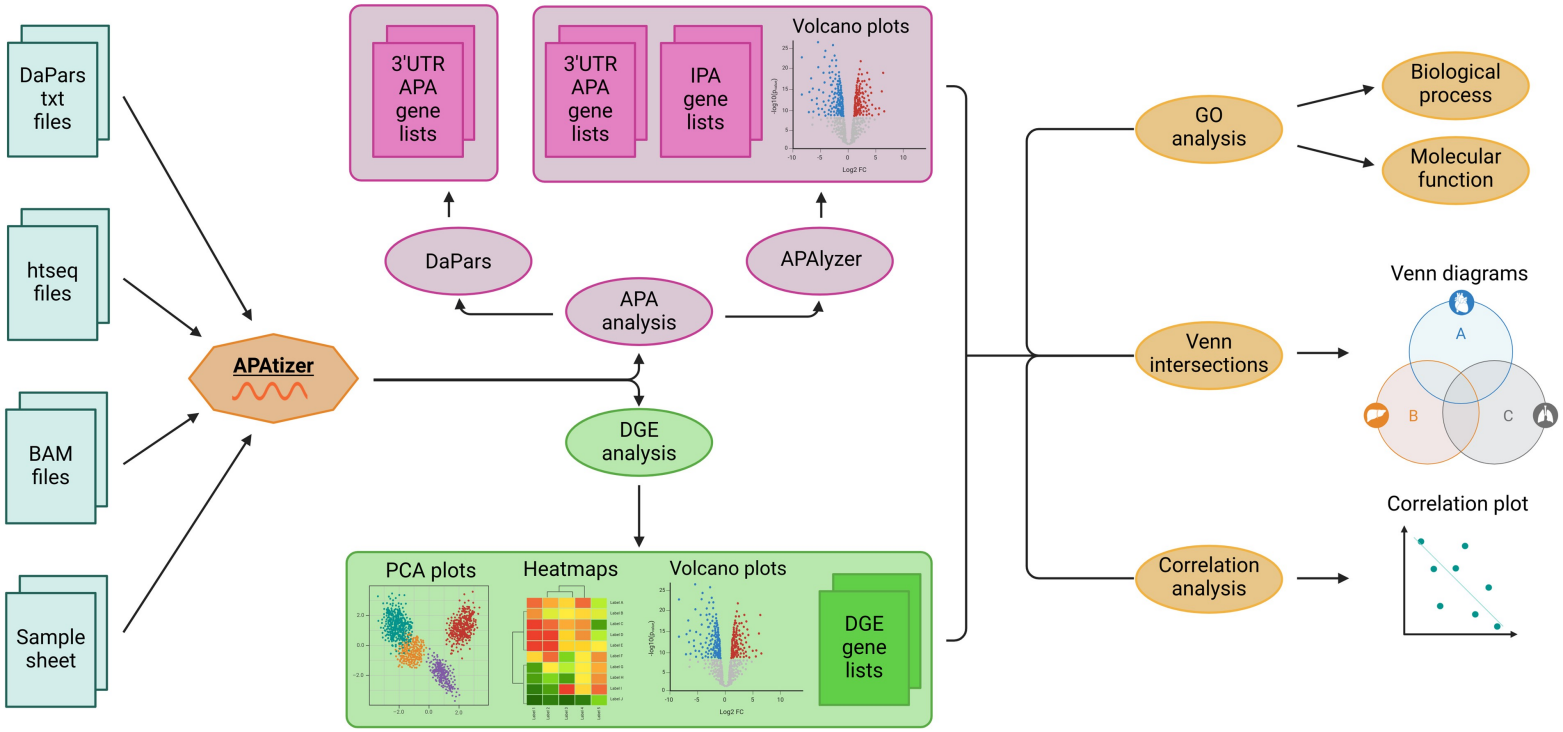
Workflow for RNA-seq analysis using APAtizer. Within the tool, the BAM files, *htseq* files obtained via htseq-count and *DaPars* bedGraph files serve as input. The user is able to perform 3ʹUTR-APA and IPA analysis, differential gene expression analysis, gene ontology analysis, Venn diagrams for intersections, and correlation analysis. In the tool’s interface, the users can obtain gene lists and informative volcano plots that can be visualized in the tool and downloaded.

APAtizer is a tool written in R using the *shiny* package (see Fig. 1). The open-source code is located at https://github.com/GeneRegulationi3S/APAtizer where a file with detailed instructions for utilization of the tool and two case studies are also available. The tool was tested in Linux under the Ubuntu 22.04 LTS (Jammy Jellyfish) distribution. The user interface is displayed upon running the R script in RStudio and the required packages are automatically installed and updated upon running the script. We also made available a docker image with the APAtizer tool with all the required packages already installed.

APAtizer has the ability to accept BAM, bedGraph, and *htseq* as input files. APAtizer can analyse both standard RNA-seq and 3ʹmRNA-seq data from Illumina and Ion Torrent technologies, supporting human and mouse genomes. In our github page we provide pre-processing scripts to create the input files for APAtizer. The tool is designed to analyse RNA-Seq data allowing the comparison between two conditions. Two case studies are described at https://github.com/GeneRegulationi3S/APAtizer. These case studies correspond to the analysis of RNA-Seq data retrieved from the Cancer Genome Atlas (TCGA) and 3ʹmRNA-Seq data (Wilton *et al.* 2023) downloaded from Gene Expression Omnibus (GEO). These examples demonstrate that APAtizer has the capability to analyse data obtained using different methodologies, such as standard RNA-Seq and 3ʹmRNA-Seq data, and from various sources. The APAtizer open-source nature ensures that it can be continually improved and adapted to answer the requirements of the research community.

## 3 3ʹUTR-APA and IPA analysis

APAtizer uses two well-known APA analysis packages: *DaPars* for 3ʹUTR-APA analysis (Feng *et al.* 2018, Li *et al.* 2021) and *APAlyzer* for 3ʹUTR-APA and/or IPA analysis (Wang and Tian 2020). *DaPars* is a widely used algorithm that identifies *de novo* APA sites from standard RNA-Seq data and *APAlyzer* is a toolkit for bioinformatic analysis of both 3ʹUTR-APA and IPA events using RNA-Seq data that compares sequencing reads in regions demarcated by high quality PASs annotated in the PolyA_DB database (Zhang *et al.* 2005). *DaPars* is capable of detecting proximal PAS usage or 3ʹUTR-APA shortening events (negative index) and distal PAS usage or 3ʹUTR-APA lengthening events (positive index) by computing the difference of alternative PAS usage between the sample pairs with a *Δ*PDUI score (Percentage of Distal polyA site Usage Index). On the other hand, *APAlyzer* compares the sample pairs and calculates a *P*-value and a relative expression difference (RED score) between the two conditions. After assigning the *P*-values and RED scores, *APAlyzer* classifies the genes into genes that display 3ʹUTR-APA shortening events, 3ʹUTR-APA lengthening events and nonsignificant events. The package considers that a gene undergoes 3ʹUTR-APA shortening if the *P*-value ≤ 0.05 and the RED score < 0, 3ʹUTR-APA lengthening if the *P*-value ≤ 0.05 and the RED score > 0, and nonsignificant if the *P*-value > 0.05. For IPA events, *APAlyzer* detects genes that show a decrease in IPA mRNAs expression (IPA downregulation), an increase in their expression (IPA upregulation) and genes whose expression present nonsignificant differences by using the same thresholds as for 3ʹUTR-APA.

In APAtizer, when using the DaPars interface, the user may input the bedgraph files originated from the DaPars algorithm and write a sample sheet inside the tool detailing the names of the files and the corresponding conditions. APAtizer then joins all the files and performs data manipulation and calculations to obtain the mean *Δ*PDUI score between the sample pairs and allows the user to obtain, search, and download lists of genes that undergo 3ʹUTR-APA shortening and lengthening events. With *APAlyzer*, the user may input the BAM files and the aforementioned sample sheet, and after the analysis is completed, can search and download gene lists for 3ʹUTR-APA events (shortening and lengthening) and IPA events (downregulation and upregulation). In addition to this, it allows for the creation of informative plots such as volcano plots and boxplots that can be visualized in the APAtizer interface and downloaded for further exploration.

## 4 Differential gene expression analysis

The differential gene expression (DGE) analysis is integrated and performed using the *DESeq2* package (Love *et al.* 2014) in the tab “DGE” in the APAtizer’s interface. *DESEq2* is a package that allows visualization and quantification of gene expression for genes that undergo downregulation and upregulation when comparing two experimental conditions.

In APAtizer, the user can obtain plots that provide useful insights in differential gene expression studies, such as PCA plots, Volcano plots and Heatmaps. The PCA plot allows the visualization of the variance between the provided conditions and might be used as a pre-processing step. The Volcano plots distinguish between downregulated (*P*-value ≤ 0.05 and log2FoldChange < −2), upregulated (*P*-value ≤ 0.05 and log2FoldChange > 2) and nonsignificant genes (*P*-value > 0.05 and/or −2 < log2FoldChange < 2). The heatmap allows the user to visualize the expression patterns between the samples of both conditions. In addition, the user can use the search box to verify if a gene of interest is located in the lists.

## 5 GO enrichment analysis

The gene ontology (GO) analysis feature in APAtizer allows the user to explore gene pathways such as biological process (BP) and molecular function (MF) of the lists of genes obtained in the APA and/or DGE analysis. For this, APAtizer uses the *cluster profiler* package (Wu *et al.* 2021) to perform over-representation analysis (ORA). The user only needs to provide one of the gene lists obtained previously and select what type of plot they desire (BP or MF). The plots can also be downloaded from the APAtizer interface.

## 6 Venn diagram intersections

APAtizer allows for the intersection of lists between a minimum of two gene lists and a maximum of five gene lists. With these intersections, one can extract the common genes between the lists as well as the specific genes to each list excluding the common ones. For visualization of the intersections, the packages *VennDiagram* (Chen and Boutros 2011) and *ggvenn* (https://github.com/NicolasH2/ggvenn) were used. The user can download the Venn diagrams displaying the intersections and the common and specific gene lists for further exploration.

## 7 Correlation analysis

APAtizer can perform correlation analysis between 3ʹUTR-APA gene lists and DGE gene lists as well as between IPA gene lists and DGE gene lists. This allows the user to analyse correlation between the gene expression and the 3ʹUTR-APA or IPA events and its significance. The tool performs a scatter plot with the line of best fit all while displaying the *P*-value and the slope for the distribution. The user can visualize said plot on the tool’s interface and download it.

## 8 Conclusions

APAtizer is a tool designed for the analysis of 3ʹUTR-APA and IPA, as well as differential gene expression, gene ontology, Venn intersections, and correlation analysis, using RNA-seq data from various sources. The main objective of APAtizer was to provide a user-friendly interface that allows all these analyses to be performed within a single platform, making it easier to retrieve important information about APA.

## Data Availability

Code and data resources for this manuscript are available in the public repository: https://github.com/GeneRegulationi3S/APAtizer.
